# Draft Whole-Genome Sequences of Two Multidrug-Resistant Salmonella enterica Serovar Senftenberg Sequence Type 14 Strains Resistant to Ciprofloxacin, Ceftriaxone, and/or Azithromycin, Isolated from Kolkata, India

**DOI:** 10.1128/mra.00978-21

**Published:** 2022-01-13

**Authors:** Priyanka Jain, Rajlakshmi Viswanathan, Gourab Halder, Sulagna Basu, Shanta Dutta

**Affiliations:** a Division of Bacteriology, ICMR-National Institute of Cholera and Enteric Diseases, Kolkata, India; b Department of Neonatology, Institute of Post Graduate Medical Education & Research and SSKM Hospital, Kolkata, India; University of Maryland School of Medicine

## Abstract

We report draft whole-genome sequences of two multidrug-resistant Salmonella enterica serovar Senftenberg sequence type 14 strains resistant to ciprofloxacin, ceftriaxone, and/or azithromycin, which were isolated from neonatal stool and goat meat in Kolkata, India. The genome characteristics, as well as the antimicrobial resistance genes, plasmid types, and integrons, are presented in this report.

## ANNOUNCEMENT

Nontyphoidal Salmonella (NTS) is one of the principal zoonotic foodborne pathogens across the globe and is implicated in causing gastroenteritis, bacteremia, and focal infections in humans (1). Antimicrobials such as third-generation cephalosporins, carbapenems, fluoroquinolones, and azithromycin have been designated critically important drugs for human use in Salmonella by the World Health Organization (2). Infections due to NTS strains that are resistant to one or more of the aforementioned drugs are on the rise (1, 3–5), which has greatly impeded the therapeutic options in humans, contributing to increased morbidity, deaths, and hospitalization costs. Early detection of such NTS strains is of utmost importance.

Of the two Salmonella enterica serovar Senftenberg strains from Kolkata, India, the first was a clinical isolate (OSS-104) recovered from stool from a preterm baby with signs of clinical sepsis who was admitted in the neonatal intensive care unit of a tertiary-care hospital in November 2009. The second strain (EVS-100) was isolated from an environmental sample (goat meat) in September 2012 during surveillance of NTS from livestock and foods of animal origin in and around Kolkata. Both isolates were serotyped at the National Institute of Cholera and Enteric Diseases (Kolkata, India) and were found to be multidrug resistant (MDR), with resistance to ciprofloxacin, ceftriaxone, and/or azithromycin, by antimicrobial susceptibility testing (6, 7). Multilocus sequence typing (MLST) of the isolates was done by PCR followed by sequencing as described earlier (8). Both isolates were typed as sequence type 14 (ST14) when sequences were submitted to the MLST database (https://enterobase.warwick.ac.uk/species/senterica/search_strains).

Whole-genome sequencing was done to determine the genetic markers of antimicrobial resistance (AMR). The genomic DNA was extracted from overnight cultures using the GeneJET genomic DNA purification kit (Fermentas Life Sciences) following the manufacturer’s instructions. The integrity of the extracted DNA was determined by gel electrophoresis. A single band of >23 kb (a lambda DNA/HindIII digest was used as a ladder) was observed, suggesting no contamination or shearing. The extracted DNA was quantified using a Qubit 4.0 fluorometer (Invitrogen; Thermo Fischer Scientific, Singapore). One microgram of quantified and unfragmented DNA was used as the input DNA. Library preparation was done using a 1D ligation kit (SQK-LSK109) without fragmentation, and the library was sequenced on the MinION platform (Oxford Nanopore Technologies [ONT], Oxford, UK) using a FLO-MIN106 flow cell (R9.4.1), following the ONT 1D native barcoding genomic DNA (with EXP-NBD104, EXP-NBD114, and SQK-LSK109) protocol (vNBE_9065_v109_revD_23May2018). The sequencing was run for 24 h. The fast5 files containing raw signal data (1.44 GB for OSS-104 and 2.22 GB for EVS-100) were generated by MinKNOW software (core v3.5.5). Base calling and adapter trimming (inbuilt) were done using ONT Guppy software (v3.3.0+ef228182). All quality reads (Q scores of >7) were segregated into a new fastq_pass folder and used for downstream analysis. Sequencing generated 14,605 reads (85.1 Mbp [read *N*_50_, 12,366 bp]) and 32,306 reads (121.8 Mbp [read *N*_50_, 10,758 bp]) for OSS-104 and EVS-100, respectively. *De novo* genome assembly was done using Canu v2.0 with default parameters for ONT sequencing (correctedErrorRate = 0.120 –nanopore-raw) (9). The genomes were annotated via the NCBI Prokaryotic Genome Annotation Pipeline (PGAP) v4.13 and Pathosystems Resource Integration Centre (PATRIC) v3.6.9 (https://www.patricbrc.org/app/Annotation) (10, 11). Acquired AMR genes and plasmids were predicted by ResFinder v4.1 (https://cge.cbs.dtu.dk/services/ResFinder) and PlasmidFinder v2.1 (https://cge.cbs.dtu.dk/services/PlasmidFinder), respectively (12, 13). The INTEGRALL database (http://integrall.bio.ua.pt) helped identify integrons (14). All analyses were performed using default parameters. The genome characteristics and genetic mechanisms of AMR in the study isolates are summarized in Table 1.

**TABLE 1:** Summary of predicted genome characteristics and genetic mechanisms of AMR in MDR *S*. Senftenberg strains from Kolkata, India

Characteristic	Data for *S*. Senftenberg strains	
	OSS-104	EVS-100
Genome size (bp)	5,159,380	5,108,715
*N*_50_ (bp)	1,637,228	2,887,881
No. of contigs	12	8
G+C content (%)	52.02	52.04
No. of coding sequences	5,212	5,038
No. of coding sequences with proteins	737	658
No. of tRNAs	84	83
No. of rRNAs	21	22
AMR genes^*a*^	*bla*_TEM-1a_, *bla*_OXA-1_, *bla*_OXA-9_, *bla*_OXA-10_, *bla*_CTX-M-15_, *cmlA1*, *catB3*, *sul1*, *aadA1*, *aac(6′)-Ib*, *aac(6′)-Ib-cr*, *aac(6')-Iaa*, *aac(3′)-IIa*, *mphA*	*bla*_OXA-1_, *bla*_OXA-10_, *bla*_CTX-M-15_, *cmlA1*, *catB3*, *sul1*, *aadA1*, *aac(6')-Ib-cr*, *aac(6')-Iaa*
Plasmid replicon	IncC (A/C_2_), Col440II	IncC (A/C_2_), Col440II
Class 1 integron^*b*^	In1251	In1251
GenBank accession no.	JAFELH000000000	JAFFQF000000000
SRA accession no.	SRR15911434	SRR15912140

a *bla*_TEM-1a_, *bla*_OXA-1_, *bla*_OXA-9_, *bla*_OXA-10_, and *bla*_CTX-M-15_ (mediating resistance to β-lactams); *cmlA1* and *catB3* (chloramphenicol resistance); *sul1* (sulfonamide resistance); *aadA1*, *aac(6')-Ib*, *aac(6')-Iaa*, and *aac(3’)-IIa* (aminoglycoside resistance); *aac(6')-Ib-cr* (plasmid-mediated quinolone resistance); and *mphA* (resistance to macrolides).

b In1251 class 1 integron (*arr2*-*cmlA1*-*bla*_OXA-10_-*aadA1* [GenBank accession number CP012902]).

Although the high error rate associated with long-read sequencing warrants a hybrid approach combining both short-read and long-read sequence data for reliable AMR allele detection, the *S*. Senftenberg assemblies derived from MinION sequencing alone in this study showed a good correlation between AMR testing and AMR gene prediction (results were confirmed by PCR and Sanger sequencing in the laboratory) and can serve as a reference from this region for genome comparison and molecular epidemiology studies.

### Data availability.

The two whole-genome shotgun projects have been deposited in DDBJ/ENA/GenBank under the BioProject PRJNA699985 with the accession numbers listed in Table 1. The versions described in this paper are JAFELH010000000 (OSS-104) and JAFFQF010000000 (EVS-100). Raw sequences have been deposited in the Sequence Read Archive (SRA) under the accession numbers listed in Table 1.
